# Optimal Stimulation Protocol in a Bistable Synaptic Consolidation Model

**DOI:** 10.3389/fncom.2019.00078

**Published:** 2019-11-13

**Authors:** Chiara Gastaldi, Samuel Muscinelli, Wulfram Gerstner

**Affiliations:** School of Computer and Communication Sciences and School of Life Sciences, École Polytechnique Fédérale de Lausanne, Lausanne, Switzerland

**Keywords:** synaptic consolidation, plasticity, LTP, stimulation frequency, bistability

## Abstract

Synaptic changes induced by neural activity need to be consolidated to maintain memory over a timescale of hours. In experiments, synaptic consolidation can be induced by repeating a stimulation protocol several times and the effectiveness of consolidation depends crucially on the repetition frequency of the stimulations. We address the question: is there an understandable reason why induction protocols with repetitions at some frequency work better than sustained protocols—even though the accumulated stimulation strength might be exactly the same in both cases? In real synapses, plasticity occurs on multiple time scales from seconds (induction), to several minutes (early phase of long-term potentiation) to hours and days (late phase of synaptic consolidation). We use a simplified mathematical model of just two times scales to elucidate the above question in a purified setting. Our mathematical results show that, even in such a simple model, the repetition frequency of stimulation plays an important role for the successful induction, and stabilization, of potentiation.

## 1. Introduction

Synaptic plasticity, i.e., the modification of the synaptic efficacies due to neural activity, is considered the neural correlate of learning (Hebb, 1949; Martin et al., 2000; Caroni et al., 2012; Nabavi et al., 2014; Hayashi-Takagi et al., 2015; Holtmaat and Caroni, 2016). It involves several biochemical mechanisms which interact on multiple timescales. The induction protocols for short-term plasticity (STP, on the order of hundreds of milliseconds) (Turrigiano et al., 1996; Markram et al., 1998) and for the early phase of long-term potentiation or depression (LTP or LTD, on the order of minutes to hours) (Levy and Stewart, 1983; Brown et al., 1989; Artola et al., 1990; Bliss and Collingridge, 1993; Markram et al., 1997; Sjöström et al., 2001) are well-established and have led to numerous models (Bienenstock et al., 1982; Gerstner et al., 1996; Song et al., 2000; Van Rossum et al., 2000; Senn et al., 2001; Shouval et al., 2002; Rubin et al., 2005; Pfister and Gerstner, 2006; Brader et al., 2007; Graupner and Brunel, 2007; Clopath et al., 2010; Gjorgjieva et al., 2011; Nicolas and Gerstner, 2016). On the other hand, various experiments have shown that the further evolution of synaptic efficacies on the timescale of hours depends in a complex way on the stimulation protocol (Frey and Morris, 1997; Dudai and Morris, 2000; Nader et al., 2000; Redondo and Morris, 2011). This phenomenon is called *synaptic* consolidation, to be distinguished from *memory* consolidation, which is believed to take place through the interaction between hippocampus and cortex and which occurs on an even longer timescale (Hasselmo, 1999; Roelfsema, 2006; Brandon et al., 2011). Such a richness of plasticity mechanisms across multiple timescales has been hypothesized to be fundamental in explaining the large storage capacity of memory networks (Fusi et al., 2005; Benna and Fusi, 2016).

**Figure 1:**
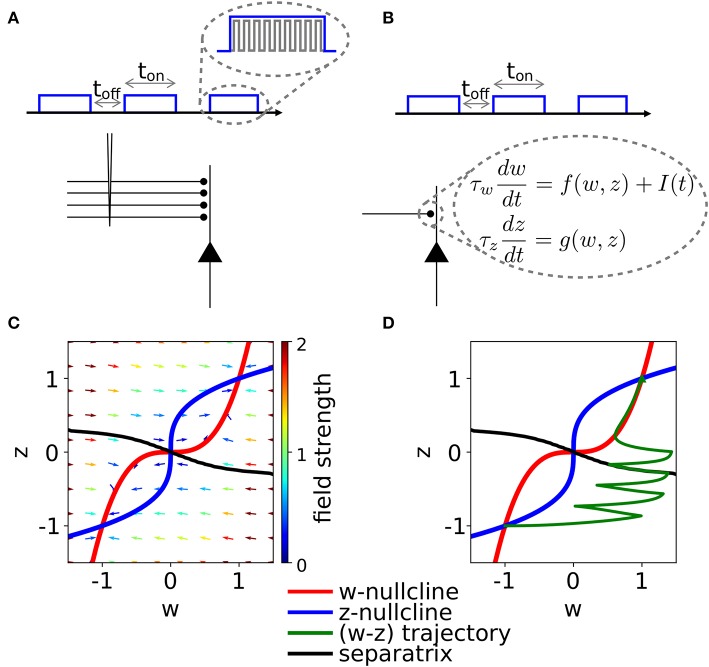
Schematic experimental setup and modeling framework. **(A)** Schematic of extra-cellular stimulation in experiments. The plasticity-inducing stimulus consists of several episodes of duration *t*_on_ with inter-episode interval *t*_off_. Zoom: Each episode contains several high-frequency pulses. **(B)** Schematic of single-synapse consolidation model. The synapse is described by a weight variable *w* with time constant τ_*w*_ and a slower consolidation variable *z* with time constant τ_*z*_ ≥ τ_*w*_. Each episode corresponds to a rectangular plasticity-inducing stimulus *I*(*t*). **(C)** Phase-plane for a specific choice of *f*(*w, z*) and *g*(*w, z*), *I*(*t*) = 0, and τ_*z*_ = 7τ_*w*_. The fixed points in (*w, z*) = (−1, −1) and (*w, z*) = (1, 1) are stable and correspond to an unpotentiated and potentiated synapse, respectively. The black line separates the basins of attraction of the two stable fixed points. **(D)** Evolution of the system dynamics in the phase-plane. The system is initialized in the unpotentiated state and it evolves under the effect of a plasticity-inducing stimulus made of three pulses.

Synaptic consolidation is often studied in hippocampal or cortical slices, in which it is induced by extra-cellular stimulation of afferent fibers with short current pulses (Frey and Morris, 1997; Sajikumar and Frey, 2004a,b). Experimental protocols are typically organized in multiple repetitions of stimulation episodes, with variable repetition frequency and duration of each episode (Figure 1A). The dependence of the consolidation dynamics on the parameters of the experimental protocol is complex and has remained elusive. Both the intra-episode pulse frequency and the inter-episode delay play an important role in determining whether a synapse gets potentiated or not after the stimulation (Kumar and Mehta, 2011; Larson and Munkácsy, 2015). Furthermore, recent evidence suggests the existence of optimal parameters to achieve consolidation (Kumar and Mehta, 2011; Larson and Munkácsy, 2015). Existing models succeeded in reproducing experimental results on early and late LTP (Clopath et al., 2008; Barrett et al., 2009; Ziegler et al., 2015; Kastner et al., 2016), by a mathematical description of the interaction of different synaptic mechanisms. However, the complexity of those models prevents a complete characterization of the dynamics, that links stimulation protocols to synaptic consolidation. Here we address the following question: why is the temporal structure of stimulation, i.e., the timing of repetitions, so important for synaptic consolidation? (Zhou et al., 2003; Kramár et al., 2012; Benna and Fusi, 2016).

We introduce a phenomenological model of synaptic consolidation (Figures 1B–D) in which, as suggested by experiments (Petersen et al., 1998; O'Connor et al., 2005; Bosch et al., 2014), both model variables are bistable. We find that, despite the simplicity of our model, potentiation of a synapse depends in a complex way on the temporal profile of the stimulation protocol. Our results suggest that not just the total number of stimulation pulses, but also the precise timing within an episode and across repetitions of episodes are important, in agreement with anecdotal evidence that changes in protocols can have unexpected consequences.

## 2. Methods

In what follows, we introduce the synaptic consolidation model that we analyze in the Results section. Since describing the details of molecular interactions inside a synapse as a system of differential equations (Bhalla and Iyengar., 1999; Lisman and Zhabotinsky, 2001) would be far too complicated for our purpose, we aim to capture the essential dynamics responsible for synaptic consolidation with an effective low-dimensional dynamical system. In this view, variables are mathematical abstractions that represent the global state of a network of biochemical molecules inside a synapse, e.g., during a transition from one metastable configuration to another (Bosch et al., 2014).

### 2.1. Choice of the Model

A one-dimensional dynamical system is not expressive enough to capture experimental data. Indeed, in a one-dimensional differential equation, it would be sufficient to know the instantaneous state of a single variable of the synapse (such as the weight) to predict its evolution, while this is not the case in experiments. As a natural step toward more complexity, we consider a general autonomous two-dimensional model

(1) $$\begin{array}{l} \frac{dw}{dt}=f\left(w,z\right)\\ \frac{dz}{dt}\;=g\left(w,z\right), \end{array}$$

where *w* represents the measured efficacy of a synaptic contact point (e.g., the amplitude of the EPSP caused by pre-synaptic spike arrival), while *z* is an abstract auxiliary variable. For simplicity, both variables will be considered unit-less. We choose the functions *f* and *g*, such that

(2) $$\begin{array}{l} \tau_{w}\frac{dw}{dt}=-K_{w}\left(w-w_{0}\right)\left(w+w_{0}\right)w+C_{w}\left(z-\frac{z_{0}}{w_{0}}w\right)+I\\ \tau_{z}\frac{dz}{dt}\;=-K_{z}\left(z-z_{0}\right)\left(z+z_{0}\right)z+C_{z}\left(w-\frac{w_{0}}{z_{0}}z\right), \end{array}$$

where (*w, z*) = ±(*w*_0_, *z*_0_) are the stable fixed points of the two-dimensional system in the presence of a fixed coupling *C*_*w*_ ≥ 0, *C*_*z*_ ≥ 0 and in the absence of a drive, i.e., *I* = 0. In our simulations, we always choose *w*_0_ = *z*_0_ = 1. For *K*_*w*_ ≠ 0 and *K*_*z*_ ≠ 0, we could divide Equation (2) by *K*_*w*_ and *K*_*z*_ to further reduce the numbers of parameters. However, we will stick to a notation with explicit *K*_*w*_ and *K*_*z*_ since we do not want to exclude the choice *K*_*w*_ = 0 or *K*_*z*_ = 0. Without loss of generality, we will choose *K*_*w, z*_ ∈ {0, 1}, i.e., either zero or unity. Note that the choice *K*_*z*_ = 0 implies that the dynamics of the auxiliary variable *z* are linear, while *K*_*z*_ = 1 implies full non-linearity. The choice of the model is explained in the next section.

### 2.2. Simplification Steps of the 2D-Dynamics

In this section we present the arguments leading from Equation (1) to (2). Readers not interested in the details may jump to the next sections. One way to tackle the very general system in Equation (1) is to perform a Taylor expansion around *w* = 0 for the first equation

(3) $$\begin{array}{l} \frac{dw}{dt}=A\left(z\right)+B\left(z\right)\cdot w+C\left(z\right)\cdot w^{2}+D\left(z\right)\cdot w^{3}+\ldots \end{array}$$

and around *z* = 0 for the second one

(4) $$\begin{array}{l} \frac{dz}{dt}=A^{\prime }\left(w\right)+B^{\prime }\left(w\right)\cdot z+C^{\prime }\left(w\right)\cdot z^{2}+D^{\prime }\left(w\right)\cdot z^{3}\ldots . \end{array}$$

An expansion up to the third order enables us to implement the bistable dynamics (Petersen et al., 1998; O'Connor et al., 2005) of single contact points. Bistability requires the system to have at least two stable fixed points at finite value. This condition cannot be met by degree 1 or degree 2 polynomials since they can have at most one *stable* fixed point. Therefore, bistability requires a polynomial of degree 3 or higher in at least one equation. To be more general, we will consider a system in which both polynomials are of degree 3. We restrict our analysis to the situation in which we have linear coupling between the two variables, of the form *A*(*z*) = *A*_0_ + *A*_1_ · *z*, *B*(*z*) = *B*, *C*(*z*) = *C*, and *D*(*z*) = *D*. Analogously, in the second equation we set $A^{\prime }\left(w\right)=A_{0}^{\prime }+A_{1}^{\prime }\cdot w$, *B*′(*w*) = *B*′, *C*′(*w*) = *C*′, and *D*′(*w*) = *D*′.

Bistability is be obtained with a negative coefficient of the third power in both equations. Before we start the analysis, we rewrite Equations (3) and (4) in a more symmetric form. To do so we proceed in three steps. (i) Assuming that the degree 3 polynomial has three real roots, we rewrite our system in the more intuitive form

(5) $$\begin{array}{l} \tau_{w}\frac{dw}{dt}=-K_{1}\left(w-w_{1}\right)\left(w-w_{2}\right)\left(w-w_{3}\right)+C_{1}z\\ \tau_{z}\frac{dz}{dt}\;=-K_{2}\left(z-z_{1}\right)\left(z-z_{2}\right)\left(z-z_{3}\right)+C_{2}w, \end{array}$$

where *C*_1_ and *C*_2_ are coupling constants and the roots *w*_1_, *w*_2_, *w*_3_ correspond to the fixed points of the equations in the uncoupled case (*C*_1_ = *C*_2_ = 0). The parameters τ_*w*_ and τ_*z*_ can be interpreted as time constants since they do not influence the location of the fixed points but only the speed of the dynamics. *K*_1_ and *K*_2_ are two positive constants that scale the whole polynomial, while *C*_1_ and *C*_2_ are positive constants that control the amount of coupling between the two variables. If we exclude the coupling terms, each equation corresponds to an over-damped particle moving in a double-well potential (Strogatz, 2014). The parameters *K*_1_, *K*_2_, τ_*w*_, τ_*z*_, *C*_1_, *C*_2_, *w*_1_, *w*_2_, *w*_3_ are simple transformations of the parameters *A*_0_, *A*_1_, *B*, *C*, *D*, $A_{0}^{\prime }$, $A_{1}^{\prime }$, *B*′, *C*′, *D*′ of the original system. For example *K*_1_ = *D*. (ii) In order to further simplify our study, we assume that in both equations one of the three roots is zero, one is positive and one negative, equally distant from zero. Following (Zenke et al., 2015), we add a plasticity induction term to the first equation that describes the drive provided by an LTP induction protocol. The equations now read

(6) $$\begin{array}{l} \tau_{w}\frac{dw}{dt}=-K_{1}\left(w-\bar{w}\right)\left(w+\bar{w}\right)w+C_{1}z+I\\ \tau_{z}\frac{dz}{dt}\;=-K_{2}\left(z-\bar{z}\right)\left(z+\bar{z}\right)z+C_{2}w. \end{array}$$

In the absence of coupling, the double well potential related to Equation (6) has minima in $w=\pm \bar{w}$, $z=\pm \bar{z}$ and a local maximum in *w* = 0 (*z* = 0). Notice that this seems to imply that a synaptic weight can take both positive and negative values, which is biologically implausible. However, this choice simplifies the calculations without loss of generality, since it is always possible to go back to a system with positive weights by applying a coordinate translation.

(iii) In the absence of a drive (*I* = 0), the system has eight free parameters, which all influence the location of the fixed points. In a final transformation step we rewrite Equation (6) such that the location of two stable fixed points becomes independent of the coupling constants *C*_1_ and *C*_2_. The reason for doing this is that the stable fixed points of the system are easier to access experimentally than other constants. In particular, the value of *w* at the stable fixed point should be related to the synaptic weight measured experimentally. We, therefore, rewrite the system in the form of Equation (2), where *w*_0_ and *z*_0_ are the absolute values of the stable fixed point and the parameters can be mapped from Equation (6) to (2), for example, *K*_*w*_ = *K*_1_ and $C_{w}=\left(K_{1}{\bar{w}}^{2}-K_{1}w_{0}^{2}\right)w_{0}/z_{0}$ and analogously for *C*_*z*_ and *K*_*z*_.

### 2.3. Nullclines and Phase-Plane Analysis

Since the system is two-dimensional, it can be studied using phase-plane analysis, following a well-established tradition in computational neuroscience (Wilson and Cowan, 1972; Ermentrout, 1996, 2002; Rinzel and Ermentrout, 1998). The fixed points of the system are graphically represented by the intersections of the nullclines (i.e., the curves defined by either $\frac{dw}{dt}=0$ or $\frac{dz}{dt}=0$), which in our system are:

(7) $$\begin{array}{ll} w-\text{nullcline:} & z=\frac{z_{0}}{w_{0}}w+\frac{K_{w}}{C_{w}}\left(w-w_{0}\right)\left(w+w_{0}\right)w-\frac{I}{C_{w}}\\ z-\text{nullcline:} & w=\frac{w_{0}}{z_{0}}z+\frac{K_{z}}{C_{z}}\left(z-z_{0}\right)\left(z+z_{0}\right)z. \end{array}$$

The maximum number of fixed points for the system in Equation (2) can be easily computed. To do so, consider a more general form of two nullclines:

(8) $$\begin{array}{ll} w-\text{nullcline:} & z=P_{n}\left(w\right)\\ z-\text{nullcline:} & w=Q_{m}\left(z\right), \end{array}$$

where *P*_*n*_(*z*) is a polynomial of degree *n* in *w* and, analogously, *Q*_*m*_(*w*) is a polynomial of degree *m* in *z* (cf. Equation 7). To find the fixed points of the system (Equation 8) we need to solve:

(9) $$\begin{array}{l} w=Q_{m}\left(P_{n}\left(w\right)\right). \end{array}$$

Equation (9) is a polynomial equation of degree *n* · *m* in *w* and therefore it allows a number of real solutions *s*, 0 ≤ *s* ≤ *n* · *m*. Applying this formula to our case, we find that we can have a maximum of nine fixed points.

In order to reduce the number of parameters from 8 to 4, we first consider the symmetric case (section 2.4) in which the two equations have the same parameters. Moreover, since we make the choice *z*_0_ = *w*_0_ = 1, the actual number of free parameters is three. In the next section, we show the effect of changing the coupling coefficients. Then, we briefly comment on the effect of the time constants and of a constant plasticity-inducing stimulus *I*. We will move to the analysis of the asymmetric cases in section 2.5.

### 2.4. Symmetric Changes of Coupling Coefficients Reveal Two Bifurcations

We study the case of symmetric coupling *C*_*w*_ = *C*_*z*_ = *C* and analyze how a change of coupling strength influences the dynamics of the system. As an aside, we note that for symmetric coupling we can define a pseudopotential (Cohen and Grossberg, 1983)

(10) $$\begin{array}{l} V(w,z)=\frac{K_{w}}{4}w^{4}+\frac{K_{z}}{4}z^{4}-\frac{1}{2w_{0}}\left(K_{w}w_{0}^{3}-z_{0}C\right)w^{2}\\ \;-\frac{1}{2z_{0}}\left(K_{z}z_{0}^{3}-w_{0}C\right)z^{2}-Cwz+I \end{array}$$

in which the dynamical variables move according to $\tau_{w}\frac{dw}{dt}=-\frac{\partial V}{\partial w}$ and $\tau_{z}\frac{dz}{dt}=-\frac{\partial V}{\partial z}$.

**Figure 2:**
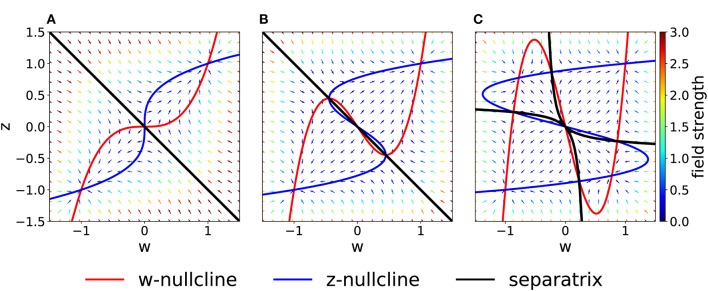
Phase-plane diagram and basins of attractions for the symmetric case with equal coupling constants, *C*_*w*_ = *C*_*z*_ = *C*. The plasticity-inducing stimulus is null, *I* = 0. **(A)** *C* = 1, phase-plane with field arrows. The color of the arrows is proportional to the field strength. *w*− and *z*− nullclines are indicated in red and blue, respectively. The line that separates the two basins of attraction is indicated in black. **(B)** Same as **(A)**, but *C* = 0.4. Compared to **(A)**, we notice the creation of two saddle points. **(C)** Same as **(A)**, but *C* = 0.2. The maximum number of fixed points is achieved. In this case we have four basins of attraction.

We fix τ_*w*_ = τ_*z*_, *K*_*w*_ = *K*_*z*_ = 1, *I* = 0, *w*_0_ = *z*_0_ = 1 and vary *C* in Equation (2). In the case *C* = 1, the system is in a rather simple regime: there are two stable fixed points in (*w, z*) = (−1, −1) and (*w, z*) = (1, 1) and a saddle fixed point at the origin (Figure 2). The basins of attraction of the stable fixed points are separated by the *z* = −*w* diagonal.

If we decrease the coupling *C*, we encounter two bifurcations. A first pitchfork bifurcation takes place at *C* = 1/2, when the two nullclines are tangent to each other in the saddle point. Beyond the bifurcation point of the coupling coefficient, we observe the creation of two additional saddle points (Figure 2B). The stability properties, the location and the basins of attraction of the other two fixed points remain unchanged, but the local field strength changes, as shown by the colored arrows. The second pitchfork bifurcation takes place at *C* = 1/3. For this coupling value, each of the two new saddle points splits into a stable fixed point and two further saddle points. Therefore, for very weak coupling we observe four basins of attractions, whose shape is shown in Figure 2C. The stability of the fixed points in (*w, z*) = (−1, −1) and (*w, z*) = (1, 1) is not affected by the bifurcations.

**Figure 3:**
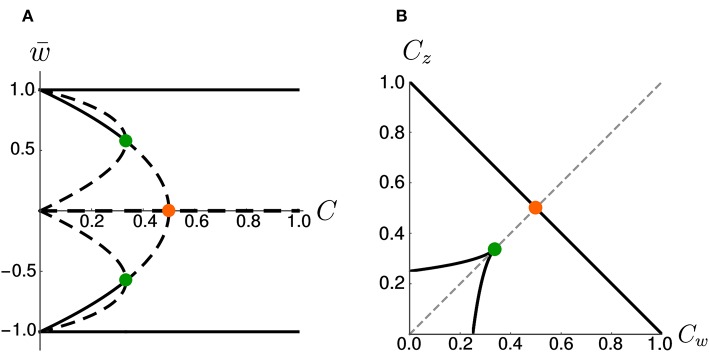
Bifurcations diagrams. **(A)** Fixed points in the symmetric case. Dashed lines indicate unstable fixed points while continuous lines indicate stable fixed points. Orange and green dots indicate bifurcation points. **(B)** Bifurcation points in the *C*_*w*_ – *C*_*z*_ plane (black) for the general (asymmetric) case. The dashed gray line corresponds to *C*_*w*_ = *C*_*z*_. The orange and green dots indicate the corresponding bifurcations in **(A)**. Note that, in **(B)**, the bifurcation at $C_{w}=C_{z}=\frac{1}{3}$ (green dot) is a degenerate point.

On the other hand, if we increase the coupling coefficient to a value *C* > 1, then the two nullclines will progressively flatten, but the location of the three fixed points is unchanged with respect to the case *C* = 1. These observations have been summarized in the bifurcation diagram of Figure 3A. We observe that there are actually three pitchfork bifurcations, but that two of them are degenerate since they happen for the same value of *C*.

### 2.5. Asymmetric Parameter Choices Shape the Basins of Attraction

**Figure 4:**
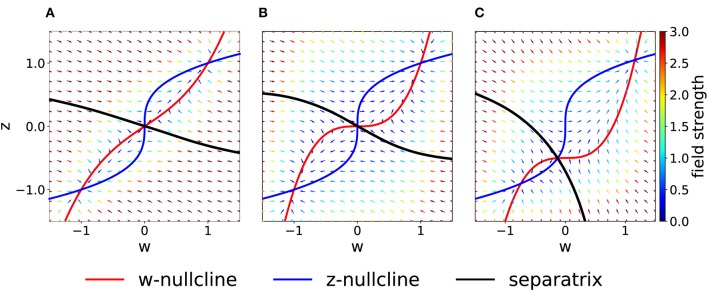
Asymmetric parameter choices. **(A)** In the case *C*_*w*_ = 3 > *C*_*z*_ = 1, the curvature of the *w*−nullcline (red) is smaller than that of the *z*−nullcline (blue) and the basins of attraction are deformed compared to Figure 2A. (τ_*z*_ = τ_*w*_ = 1) **(B)** For τ_*z*_/τ_*w*_ = 3 and *C*_*w*_ = *C*_*z*_ = 1, nullclines are not affected (compare to Figure 2A) but the basins of attraction are. **(C)** For *I* = 0.5 (all other parameters set to 1), the basin of attraction of the fixed point at (−1, −1) is smaller than of the fixed point at (1, 1).

As a more general case, we consider asymmetric coupling *C* or timescale τ. When the coupling coefficients are asymmetric, we can plot the position of the bifurcation points in the *C*_*w*_—*C*_*z*_ plane (Figure 3B). The choice *C*_*w*_ = *C*_*z*_ of the previous section corresponds to the dashed gray line. We notice that in the asymmetric case it is possible to have three distinct bifurcations (for example, we can fix *C*_*w*_ = 0.3 and decrease *C*_*z*_, from 1 to 0). We find that, for *C*_*w*_ + *C*_*z*_ > 1, the number of fixed points is always three and no bifurcation is possible. On the other hand, if *C*_*w*_ + *C*_*z*_ < 1, the system enters in the regime with minimum five fixed points. Moreover, we can analytically compute the bifurcation value of one coupling constant, given the other. An asymmetric choice *C*_*w*_ ≠ *C*_*z*_ influences the shape of the basins of attraction (Figure 4A).

If we keep *C*_*w*_ = *C*_*z*_ but consider instead τ_*z*_ > τ_*w*_, the system in Equation (2) may be interpreted as two different molecular mechanisms that act on different timescales. For example, the variable *z* can be interpreted as a tagging mechanism or a consolidation variable while *w* is the weight variable or amplitude of a post-synaptic potential. A comparison of Figure 2A and Figure 4B shows that the changes in τ do not affect the nullclines but change the flow field and the basin of attraction.

Another way by which we can introduce asymmetry in the system is by adding a plasticity-inducing stimulus *I*. It follows from Equation (7) that a value *I* > 0 will cause a down shift of the *w*−nullcline. The case of *C*_*w*_ = *C*_*z*_ = 1, τ_*w*_ = τ_*z*_ = 1 s, *K*_*w*_ = *K*_*z*_ = 1 and *I* > 0 is shown in Figure 4C. A plasticity-inducing stimulus *I* > 0 also implies a reduction of the basin of attraction of the lower stable fixed point in favor of an increase of the basin of attraction of the upper stable fixed point. For high values of *I*, the basin of attraction of the lower fixed point disappears via a steady state bifurcation. Therefore, when *I* > 0 is large enough, the system is forced to move to the upper fixed point that can be interpreted as a potentiated state of the synapse. Analogously, when *I* < 0, the attraction basin of the lower fixed point is enlarged and leads, eventually, to a bifurcation in which the upper fixed point and the saddle point are lost.

A possible generalization of the model would be to consider the coupling coefficients *C*_*w*_ and *C*_*z*_ as dynamical variables, as it has been explored in previous work (Ziegler et al., 2015). In these models, the coupling parameters *C*_*w*_ and *C*_*z*_ of the two dynamical variables alternates between *C*_*w*_ = 0 and *C*_*z*_ = 1 or *C*_*w*_ = 1 and *C*_*z*_ = 0, implementing a write-protection mechanism. The price we pay is the introduction of additional differential equations and parameters for the dynamics of the coupling coefficients. In the specific implementation of Ziegler et al. (2015), the dynamical coupling is controlled by a low-pass filter of the plasticity-inducing stimulus *I* and the concentration of neuromodulators on plasticity.

### 2.6. Numerical Simulations

All figures were obtained using Python 2.7, except for the bifurcation plot in Figure 3, which was created with Wolfram Mathematica. In the phase-plane plots, the separatrix between the basins of attraction was obtained doing a mesh-grid search: we initialized the dynamical system (Equation 2) in each point of a 100 × 100 grid in the *w, z* space (*w, z* ∈ [−1.5, 1.5]) and checked to which stable fixed point it converges. Therefore we interpolated the separation line. The trajectory of the system in the phase-plane was obtained by solving the system in Equation (2) using the Runge-Kutta 4 method with integration step *dt* = 0.01. In **Figures 6**, **7**, we inject an external stimulus into the dynamical equations. The system trajectory is always initialized in the depotentiated state (−1, −1) and the simulation is stopped when the trajectory enters into the basin of attraction of the potentiated state (1, 1). The position of the stable fixed points depends on the choice *w*_0_ = *z*_0_ = 1, which we made for simplicity. In fact, we can remap the values of the synaptic weight *w* the desired (positive range) with an affine transformation, without loss of generality.

## 3. Results

The two-dimensional model, introduced Methods section, predicts a complex dependence of the synaptic consolidation dynamics upon the parameters of the experimental protocol. This complex dependence has similarities with the behavior observed in experiments (Sajikumar et al., 2005; Larson and Munkácsy, 2015; cf. Figure 1). First, we describe how we abstract the experimental protocol into a time-dependent plasticity-inducing stimulus *I*(*t*). Then, we show the response of our model to different stimulation protocols. In our model, the plasticity-inducing stimulus *I*(*t*) drives the synaptic weight *w* via a non-linear equation characterized by a time constant τ_*w*_. The weight *w* is coupled to a second variable *z* with time constant τ_*z*_ (Equation 2). The variable *z* is an abstract description of the complex metastable states (potentiated or unpotentiated) caused by consolidation (Redondo and Morris, 2011; Bosch et al., 2014). After an analysis of a single rectangular stimulation (one episode), in section 3.2, we will move to the more realistic case of repetitive stimulation across multiple episodes. Throughout the results section, we will focus on synaptic potentiation. Since the self-interaction term in Equation (2) is symmetric with respect to *w* = *z* = 0, synaptic depression of a potentiated state is the mirror image of synaptic potentiation of a unpotentiated state.

### 3.1. Abstraction of the Stimulation Protocol

In their seminal work, Bliss and Lømo (1973) showed that repeated high-frequency stimulation of afferent fibers can lead to long-lasting synaptic potentiation. In later work it was shown that low-frequency stimulation can lead to long-lasting synaptic depression (Bashir and Collingridge, 1994). In order to keep the analysis transparent, we use a time-dependent, real-valued quantity *I*(*t*) as an abstraction for such experimental protocols. In what follows, we will refer to *I*(*t*) as to the plasticity-inducing stimulus. Note that, we do not perform an explicit mapping from the electrical current used in LTP experiments for the stimulation of pre-synaptic fibers onto the plasticity-inducing stimulus *I*(*t*) that influences the dynamics of Equation (2). A precise mapping would require additional assumptions on (i) how extra-cellular stimulation triggers axonal spikes in multiple fibers, (ii) how pre-synaptic spike arrivals cause post-synaptic firing and (iii) how pre- and post-synaptic neural activity leads, potentially via a Hebbian model, to the induction of early-LTP. This means that, in principle, the model's dynamics is rich enough to reproduce the four classical synaptic-consolidation experiments (Frey and Morris, 1997; Nader et al., 2000), however, we would need to set at least four free parameters, corresponding to the amplitudes of the external input *I*, needed for strong and weak LTP and LTD. Instead, we model a set of extra-cellular high-frequency pulses as a single rectangular plasticity-inducing stimulus of positive amplitude (Figure 1B). The larger the stimulation frequency, the larger the amplitude of *I*(*t*). Analogously, a set of extra-cellular current pulses at low frequency is modeled as a single negative rectangular plasticity-inducing stimulus. The compression of multiple extra-cellular pulses into a single rectangular episode *I*(*t*) is justifiable since the time between single pulses, even in the case of low-frequency stimulation, is very short compared to the timescale of plasticity. This implies that multiple short pulses in experiments can be well approximated by a single episode, described by one prolonged rectangular stimulus in our model (Figures 1A,B). In agreement with well-established plasticity models (Bienenstock et al., 1982; Senn et al., 2001; Pfister and Gerstner, 2006; Clopath et al., 2010; Gjorgjieva et al., 2011), we use *I* > 0 to describe a high-frequency stimulation since a positive *I* leads to potentiation (see section Methods and Figure 4C). Conversely, a negative *I* favors depotentiation. On the other hand, experiments that involve global variables, such as cross-tagging (Sajikumar and Frey, 2004a,b), can not be explained by our model.

### 3.2. One Episode

**Figure 5:**
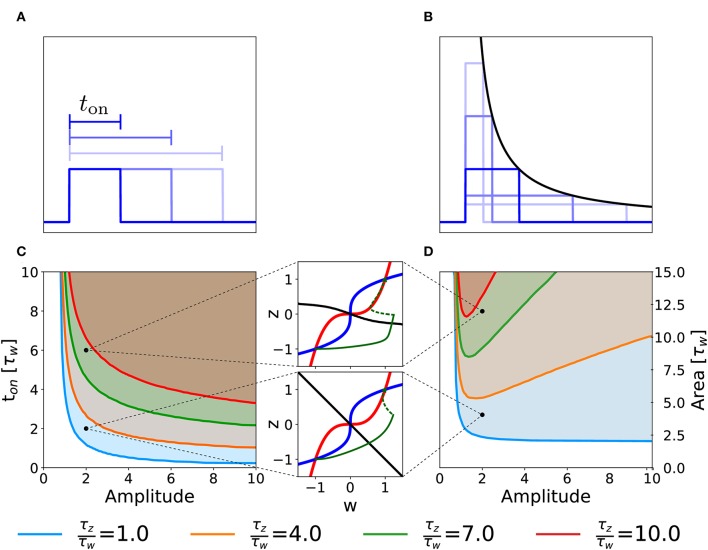
Potentiation during a single episode. Different curves correspond to different ratios of the time constant τ_*z*_ and τ_*w*_ in Equation (2). **(A)** Schematic representation of single-episode stimulations, corresponding to different choices of *t*_on_. **(B)** Schematic representation of different single-episode stimulations with constant area. The black line is proportional to 1/amplitude in order to stress that all pulses have the same area. **(C)** Separation curves between regions of successful or unsuccessful potentiation as a function of amplitudes and duration *t*_on_ of a the plasticity-inducing stimulus. The initial state is always the unpotentiated synapse (*w* = *z* = −1). The shaded region of the parameter space is the one in which the synapse gets potentiated. **(D)** Same as **(C)** but as a function of amplitude and area of the stimulus during the episode. The two insets show examples of trajectories (green lines) in the phase-plane for two different parameters choices. The solid green lines represent the dynamical evolution of the system during the application of the external stimulus, while the dotted green line shows the relaxation of the system to a stable fixed point after the stimulation. Red: *w*-nullcline; blue: *z*-nullcline; black: separatrix. The parameters that are not specified in the figure are: *C*_*w*_ = *C*_*z*_ = 1, *I* = 0.

We consider the case in which our two-variable synapse model is stimulated with a single rectangular plasticity-inducing stimulus *I*(*t*) of variable amplitude and duration *t*_on_ (Figure 5A). Experimentally, this would correspond to single-episode, high-frequency protocols of variable stimulation intensity (i.e., pulse frequency) and duration. For each choice of duration and amplitude, we initialize the system in the unpotentiated state, defined by the initial value (*w, z*) = (−1, −1) and we numerically integrate the system dynamics until convergence. We then measure the final state of the synapse, i.e., whether it converged to the potentiated or returned to the unpotentiated state. In Figure 5, we plot the curve that separates the region of the parameter space that yields potentiation (shaded area) from the one that does not. Different curves correspond to different time constants τ_*w*_ and τ_*z*_ of the synaptic variables *w* and *z* in Equation (2).

Figure 5C illustrates a rather intuitive result, i.e., if the amplitude of the plasticity-inducing stimulus is increased, the duration needed for potentiation decreases. Moreover, if the amplitude is too small, we cannot achieve potentiation, even for an infinite pulse duration. The limit of infinite pulse duration is in the following called the “DC” limit. The effect of DC-stimulation can be easily understood from a phase-plane analysis (Figure 4). Indeed, the introduction of a constant term *I* > 0 in the *w* equation (DC term), yields a shift in the *w*-nullcline vertically downward. However, if the term is too small to cause the loss of the low fixed point, potentiation cannot be achieved (Figure 4C).

The separation curves in Figure 5C indicate that the minimal duration of an episode necessary for potentiation decreases as the intensity of the plasticity-inducing stimulus increases. We wondered whether the relevant parameter for potentiation is the area under the rectangular plasticity-inducing stimulus. To study this, we performed a similar analysis, with the amplitude of the plasticity-inducing stimulus and its area as independent variables. For each choice of area and amplitude, the duration of the episode is given by *t*_on_ = area/amplitude (see Figure 5B). The results are shown in Figure 5D. If there were a regime in which the relevant parameter is the area of the pulse, then the curve separating parameters of successful from unsuccessful potentiation would be horizontal. However, we find a near-horizontal curve only for τ_*z*_ = τ_*w*_, limited to the high-amplitude region. For τ_*z*_ > τ_*w*_ we find the existence of an optimal value of the amplitude that yields potentiation with the minimal area. If we increase the amplitude beyond this optimal value, the necessary area under the stimulus curve *I*(*t*) starts to increase again.

In order to understand this effect, we look again at the phase-plane, in particular at the dependence of the separatrix on the timescale separation. In the limit in which the amplitude goes to infinity and the duration goes to 0 while the area of the whole plasticity-inducing stimulus stays the same, the stimulus can be described by a Dirac-δ function. In Figure 5D, we can see that, if τ_*z*_ ≫ τ_*w*_, the separatrix tends to an horizontal line for *w* ≫ 1. Since a δ-pulse input is equivalent to an instantaneous horizontal displacement of the momentary system state in the phase-plane, a single δ-pulse cannot bring the system across the separatrix. The δ-pulse stimulation is, of course, a mathematical abstraction. In a real experimental protocol, such a stimulation can be approximated by a short episode of very intense high frequency stimulation. Due to the necessary finite duration of an episode, the system response in the phase-plane will not be a perfectly horizontal displacement. However, achieving potentiation with short pulses can still be considered as difficult, because it would require a disproportionately large stimulation amplitude.

Our findings highlight the fact that changing parameters, such as the ratio of τ_*w*_ and τ_*z*_, gives rise to different behaviors of the model in response to changes in the stimulation protocols. We may use this insight to design optimal experimental protocols for single-episode plasticity induction. In particular, a model with timescale separation would predict the existence of an optimal stimulus intensity for which the total stimulus area necessary for potentiation is minimized. We emphasize that any model where consolidation works on a timescale that is slower than that of plasticity induction will exhibit timescale separation and be therefore sensitive to details of the stimulation protocol.

### 3.3. Repeated Episodes

**Figure 6:**
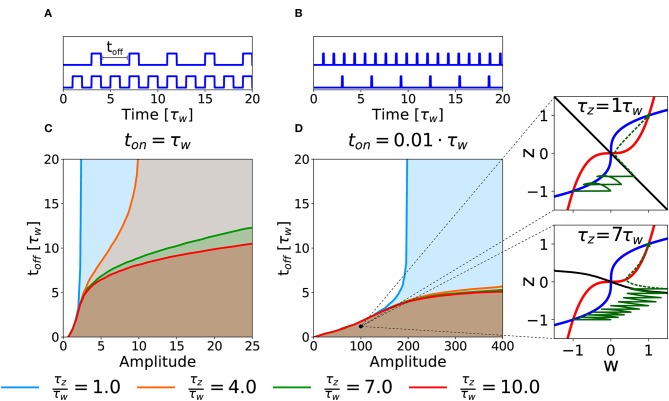
Potentiation with repeated episodes. **(A)** Schematic representation of stimulation protocols characterized by different *t*_off_, while *t*_on_ = τ_*w*_ is fixed. **(B)** Schematic representation of stimulation protocols with *t*_on_ = 0.01τ_*w*_. **(C)** Potentiation region for stimulation with long episodes for fixed *t*_on_ = τ_*w*_. The curves for different ratios τ_*z*_/τ_*w*_ (see color code) indicate the separation between the region that yields potentiation (shaded) and the region that does not (white) as a function of intensity of stimulation in each episode (amplitude) and inter-episode interval (*t*_off_). **(D)** Potentiation regions for protocols with shorter episode *t*_on_ = 0.01τ_*w*_. The potentiation region is shaded. The two insets show examples of trajectories (green lines) in the phase-plane for the same choice of stimulation parameters but different timescale separation. The solid green lines represent the dynamical evolution of the system during the application of the external stimulus, while the dotted green line shows the relaxation of the system to a stable fixed point after the end of the stimulation protocol. The parameters that are not specified otherwise are: *C*_*w*_ = *C*_*z*_ = 1, *I* = 0.

As a second case, we consider the potentiation of a synapse induced by repetitions of several stimulation episodes. In an experimental setting, this type of stimulation would correspond to several episodes of high-frequency stimulations, characterized by three parameters: the intensity of stimulation during each episode (amplitude), the duration (*t*_on_) of each episode and the inter-episode interval, *t*_off_ (cf. Figures 6A,B). To keep the analysis transparent we apply a number of repetitions large enough to decide whether potentiation is successful or not given the three parameters. Notice that if *t*_off_ = 0 we are back to the DC stimulation as defined in the previous section.

The curves in Figures 6C,D show the separation between parameters that lead to successful potentiation (shaded) or not (white) in the amplitude-*t*_off_ space for fixed values of *t*_on_ and for different τ_*z*_/τ_*w*_ ratios. In Figure 6C, we fix *t*_on_ = τ_*w*_. We observe that, at least for low timescale ratios, it exists an amplitude above which the synapse gets potentiated independently of *t*_off_, which suggests that, for this intensity of the stimulation, the potentiation happens during the first episode. The amplitude necessary to obtain potentiation in one pulse, however, increases rapidly with the τ_*z*_/τ_*w*_ ratio (see section 3.2). On the other hand, if the value of *t*_off_ is small enough (i.e., for high repetition frequency), potentiation can be achieved with smaller amplitudes and the timescale ratio is less important (notice the superimposed lines in the bottom left part of the plot). If we decrease the pulse duration to *t*_on_ = 0.01τ_*w*_, we obtain qualitatively similar separation curves, but potentiation now requires much larger values for the amplitude of the plasticity-inducing stimulus (see Figure 6B), than *t*_on_ = τ_*w*_ (see Figure 6A). Importantly, in the case of timescale separation (e.g., τ_*z*_ = 7τ_*w*_) several repetitions are needed before the consolidation variable *z* has sufficiently increased so that the synapse state crosses the separatrix (Figure 6, insert).

**Figure 7:**
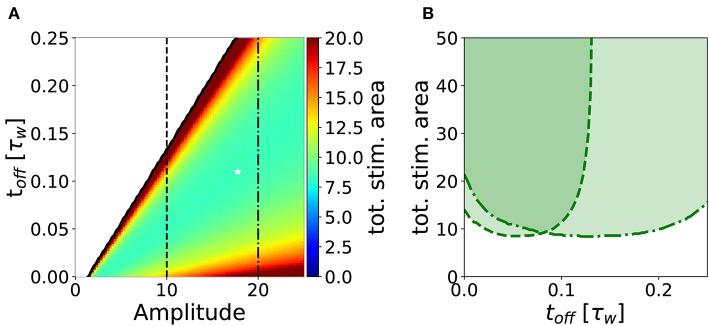
Stimulation effort needed to achieve potentiation for τ_*z*_/τ_*w*_ = 7 and *t*_on_ = 0.01τ_*w*_. **(A)** The potentiation domain (shaded region in Figure 6) is colored proportionally to the stimulation area needed to achieve potentiation with a repetitive pulse stimulus. The minimum stimulation area is ≃ 8.34, it is indicated by the white star and corresponds to the parameters values *t*_off_ = 0.11τ_*w*_, amplitude = 17.75 and 47 pulses. **(B)** Slices of the diagram in **(A)** for amplitude = 10 (dashed line) and for amplitude = 20 (dash-dotted line) are plotted against *t*_off_. One can notice that for a fixed stimulation amplitude, there is an optimal repetition frequency $f=\frac{1}{t_{\text{on}}+t_{\text{off}}}$ that minimizes the stimulus area required to achieve potentiation. The parameters that are not specified otherwise are: *C*_*w*_ = *C*_*z*_ = 1, *I* = 0.

In analogy to the analysis performed in section 3.2, we search for an optimal stimulation protocol in the case of repeated episodes. In order to allow a direct comparison between single and repetitive episodes, we measured the total area under the stimulation curve *I*(*t*) in the repetitive episode scenario, limited to the minimal number of episodes sufficient to achieve potentiation. In Figure 7A, we show the minimum stimulation area (number of episodes times the area of each rectangular plasticity inducing stimulus) required to achieve potentiation, as a function of the amplitude and the frequency of the stimulus for strong timescale separation (τ_*z*_/τ_*w*_ = 7). We notice that the minimum stimulation area (white star) corresponds to *t*_off_ ~ 10*t*_on_, i.e., the waiting time between episodes is ten times long than each episode. In real experimental conditions, however, it might be difficult to control the intensity of the stimulation. For this reason, we consider a fixed intensity (e.g., amplitude *I* = 10 in Figure 7A) and vary the inter-episode time *t*_off_. We find that there exists an optimal stimulation frequency to obtain potentiation with minimal total area (see Figure 7B). These results highlight two main facts: (i) for many stimulation intensities (only two are shown in the graph), one can find an optimal repetition frequency, (ii) there is a broad region in the parameter space (*t*_on_, amplitude, and area) where the number of pulses needed to achieve consolidation is constant. Indeed, the broad region around the minima in Figure 7B (fixed amplitude and *t*_on_) where the area is approximately constant corresponds a constant number of pulses (*n*_pulses_ = area/*t*_on_).

## 4. Discussion

We introduced and analyzed a minimal mathematical model of synaptic consolidation, that consists of two ODEs with linear coupling terms and cubic non-linearity. Since it is a two-dimensional model, the system can be studied using phase-plane techniques. While our model can have up to four stable fixed points, we focused on the case of two stable fixed points, to allow the physical interpretation of the fixed points as an unpotentiated or potentiated synapse. The weight variable *w* should be seen as the bistable building block of complex synapses. While there is evidence that the potentiation of a single synapse is a all-or-none process (Petersen et al., 1998; O'Connor et al., 2005; Bosch et al., 2014), recent results challenge this view in favor of a modular structure of the synapse (Lisman, 2017). Either way, it is possible to identify a bistable basic element of the synapse.

We showed that our minimal model responds to stimulation protocols in a non-trivial way: we quantified the total stimulation strength by the stimulus area defined as duration times intensity, where intensity is a combination of intra-episode frequency and current amplitude of extra-cellular pulses. We found that the minimal stimulus area necessary to induce potentiation depends non-monotonically on the choice of stimulus parameters. In particular, we found that, for both single-episode and multiple-episode stimulation, it is possible to choose the stimulation parameters (intensity, duration, and inter-episode frequency) optimally, so as to minimize the stimulus area (Figures 5, 7 and Table 1). Figure 7 can be used to compare the minimum stimulation area needed to achieve potentiation in a single episode (corresponding to the choice *t*_off_ = 0) to the case of repetitive pulses (*t*_off_ ≠ 0). We conclude that, for a fixed stimulation area, stimulation over several episodes is advantageous to achieve potentiation, in agreement with some widely used protocols (Larson and Munkácsy, 2015). The effect is stronger if the consolidation variable *z* is slow compared to the weight variable (τ_*z*_ ≫ τ_*w*_). Note that in experiments it is often impossible to have a fine control on the stimulation amplitude: extra-cellular stimulation of fibers must be strong enough to excite the post-synaptic neuron, but there is no control of the post-synaptic firing rate, which could undergo adaptation or exhibit other time-dependent mechanisms. In summary, the existence of an optimal stimulation frequency is the direct consequence of two very fundamental synaptic properties: (i) the bistability of a synaptic basic element, and (ii) the time scale separation between the internal synaptic mechanisms.

**Table 1:** Parameter values used in Figures 5–7 unless otherwise indicated in figures captures.

**Parameter**	**Figure 2**	**Figure 4**	**Figure 5**	**Figure 6**	**Figure 7**
*C*_*w*_	1	1	1	1	1
*C*_*z*_	Variable	Variable	1	1	1
*w*_0_	1	1	1	1	1
*z*_0_	1	1	1	1	1
τ_*w*_	1	1	1	1	1
τ_*z*_	1	Variable	Variable	Variable	7
*dt*	0.01	0.01	0.01	0.01	0.01

The minimum of the total stimulus area is particularly pronounced in the regime of strong separation of timescale (τ_*z*_ ≫ τ_*w*_), which is the relevant regime in view of the experimental consolidation literature which suggests multiple consolidation mechanisms with a broad range of timescales (Bliss and Collingridge, 1993; Reymann and Frey, 2007). Assuming that the timescale τ_*w*_ is on the order of a few seconds, as suggested by some plasticity induction experiments at the level of single contact points (Petersen et al., 1998), we can interpret a short stimulation episode of duration 0.01 · τ_*w*_ ~ 20 ms as a burst of few pulses at high frequency. For example, one particularly interesting protocol is the theta burst stimulation, where each burst consists of 4 pulses at 100 Hz corresponding to a burst of 30 ms duration (Larson and Munkácsy, 2015). Assuming that this stimulation does not correspond to an extremely small amplitude value (a reasonable assumption since the experimentalists want to induce LTP), our model predicts an optimal frequency (see Figure 7) on the order of *t*_off_ = 0.11τ_*w*_ ~ 200 ms, which is in rough agreement with the experimental protocols where theta bursts are repeated every 200 ms (Larson and Munkácsy, 2015). When comparing the optimal stimulation frequency obtained by our model to experimental data, we should keep in mind that in experiments timing effect come from different sources. In Larson and Munkácsy (2015), the key factor that determines the optimal stimulation protocol is the feed-forward inhibition. Moreover, in Sajikumar et al. (2005), Kumar and Mehta (2011), and Larson and Munkácsy (2015) the position of the stimulation (apical vs. basal) plays a fundamental role, together with priming of NMDA channels. Finally, the fraction of NMDA vs. AMPA receptors is another fundamental element. None of these factors is taken into account in our simplified model.

We have described the simplified dynamics of a bistable basic element of synaptic consolidation (which can be interpreted as a single contact point or a synaptic sub-unit). However, in most experiments, we can only observe the collective effect of many such elements together (Malenka, 1991; Bliss and Collingridge, 1993). Such a collective effect can be interpreted as the average number of potentiated contact points. For a detailed comparison between our model and these experiments, it would be needed to simulate the dynamics of the pre- and post-synaptic neuron groups and of their contact points, in order to obtain an average quantity that can be compared with the continuous change of EPSP observed in experiments. Such approach has been taken in Ziegler et al. (2015) and it requires several assumptions, among others the specification of the dependence of the plasticity induction current *I* on the pre- and post-synaptic activity, the parameters of the two populations and possible recurrent interactions (see also 3.1) (see Supplementary Material, for a qualitative comparison). For these reasons, such a comparison goes beyond the aim of this work. Moreover, since the model describes a single synaptic contact, it cannot be applied to more complex experiments that involve cross-tagging, where effects of protein synthesis are shared between several synapses (Sajikumar and Frey, 2004b). On the other hand, our results highlight the fact that our model shares similar response properties with the population-averaged quantities measured in experiments, such as its sensitivity to the stimulation frequency and the preference for multiple repetitions. Altogether, these findings suggest that our model possesses the necessary dynamical repertoire to reproduce some of the experimental results (such as Malenka, 1991; Bliss and Collingridge, 1993).

Using our model we can only make some qualitative predictions on experimentally measurable quantities. For example, by comparing Figures 6C,D, we can see that the optimal stimulation parameters change when varying the episode duration *t*_on_. More precisely, our model predicts that for shorter *t*_on_ the optimal stimulus requires a large stimulus intensity during each episode.

The proposed framework is related to a number of previous modeling approaches to synaptic consolidation. In particular, the memory formation in networks of excitatory and inhibitory neurons in Zenke et al. (2015) is based on a synaptic plasticity model with a linear weight variable and a slower consolidation variable, corresponding to a choice of *K*_*w*_ = 0 in Equation (2) of our model. If we exploit this relation between the two models, the coupling term *C*_*w*_ should depend on the post-synaptic activity. Such a time-dependent coupling coefficient is similar to the gating variable in the write-protected model (Ziegler et al., 2015). The write-protected model (Ziegler et al., 2015) can be considered as a three-dimensional generalization of our framework. In our model the weight variable *w* is directly coupled to the consolidation variable *z* whereas in the write-protected model *w* is coupled to an intermediate tag-related variable which is then coupled to *z*.

The dynamical understanding of the interplay between stimulation protocol and autonomous dynamics gained here by studying the two-dimensional system can be also applied to a three-(or higher-)dimensional generalization, under the assumption that coupling exists only between pairs of variables and that there is timescale separation. Using such a multi-dimensional generalization, it would be possible to explain a much larger set of experimental results. In addition, the model presented in Ziegler et al. (2015) features coupling coefficients that are dynamically adjusted as a function of the induction protocol itself. A change of coupling *C* makes a model at the same time more expressive and harder to analyze (cf. section 2.3).

The cascade model (Fusi et al., 2005) can be related to the model in the present paper by introducing several slow variables *z*_1_, …, *z*_*n*_ with time constants τ_1_, …, τ_*n*_. The coupling from *k* to *k* + 1 is analogous to the coupling of *w* to *z* in Equation (2). Even though this extended model and the cascade model share the concept of slower variables, there are some important differences between the two. First, the cascade model (Fusi et al., 2005) is intrinsically stochastic, i.e., the stochasticity due to spiking events is combined with the stochasticity of plasticity itself. Second, the transitions among states in the cascade model are instantaneous (Fusi et al., 2005). In our framework instead, even though there are discrete *stable* states, the transitions need some time to happen and this is exactly why the frequency of a repetitive stimulus matters in our model.

Similarly to the cascade model (Fusi et al., 2005), the “communicating vessels” model (Benna and Fusi, 2016) relies on multiple hidden variables. However, in contrast to the cascade model (Fusi et al., 2005), the dynamics in the “communicating-vessels” model are determined by continuous variables that obey continuous-time differential equations (Benna and Fusi, 2016). If we truncate the “communication-vessels” model to a single hidden variable, the resulting dynamics fall into our framework, with the simple choice *K*_*w*_ = *K*_*z*_ = 0. Extensions to multiple hidden variables with progressively bigger timescales is possible analogously to our discussion above. Indeed experimental results show that the internal bio-chemical mechanisms of the synapse are characterized by different timescales (Reymann and Frey, 2007; Bosch et al., 2014).

Similar to the cascade model, the “state based model” proposed in Barrett et al. (2009), consist of synapses whose state can shift from e-LTP to l-LTP according to some transition-rates. The model captures two internal mechanisms (tagging and anchor for AMPAR). The probability that a particular synapse is in a specific state is a continuous quantity that depends on the transition probabilities. The main similarity to our model is the presence of a bistable basic synaptic element.

Finally the synaptic plasticity model proposed in Shouval et al. (2002) proposes a non-linear dynamics for the synaptic weights, similarly to our model. The main goal of Shouval et al. (2002) is to relate the amount of NMDAR with the Calcium level in the synapse. However, their model is not bistable and no attempt is made to capture the internal synaptic state.

To summarize, our model focuses on a single transition using two variables. If these variables have different intrinsic timescales, the temporal pattern of the stimulation protocol plays a crucial role. We believe that these insights are applicable beyond our two-variable model in situations where multiple variables covering multiple timescales are pair-wise coupled to each other. This includes well-known consolidation models such as the cascade model (Fusi et al., 2005), the communicating vessels model (Benna and Fusi, 2016), and the write-protected synapse model (Ziegler et al., 2015).

## Data Availability Statement

All datasets analyzed for this study are included in the article/Supplementary Material.

## Author Contributions

All authors contributed to the conception of the study. CG implemented the code needed to produce all figures, except for Figure 3. SM performed the bifurcation analysis presented in Figure 3 and contributed to the revision and improvement of the figures. CG and SM wrote the first draft of the manuscript. All authors contributed to correcting and improving the manuscript. All authors contributed to manuscript revision, read, and approved the submitted version.

### Conflict of Interest

The authors declare that the research was conducted in the absence of any commercial or financial relationships that could be construed as a potential conflict of interest.
