# Supplementary material for: Optimal Stimulation Protocol in a Bistable Synaptic Consolidation Model
Source: Front Comput Neurosci. 2019 Nov 13;13:78. doi: 10.3389/fncom.2019.00078 (PMC6874130; doi:10.3389/fncom.2019.00078)
Supplement: Supplementary file 1 [file Data_Sheet_1.PDF]

Optimal stimulation protocol in a bistable synaptic consolidation model Supplementary Material

Chiara Gastaldi [1], Samuel Muscinelli [1] and Wulfram Gerstner[1]

[1]School of Computer and Communication Sciences and School of Life Sciences École polytechnique fédérale de Lausanne, Switzerland

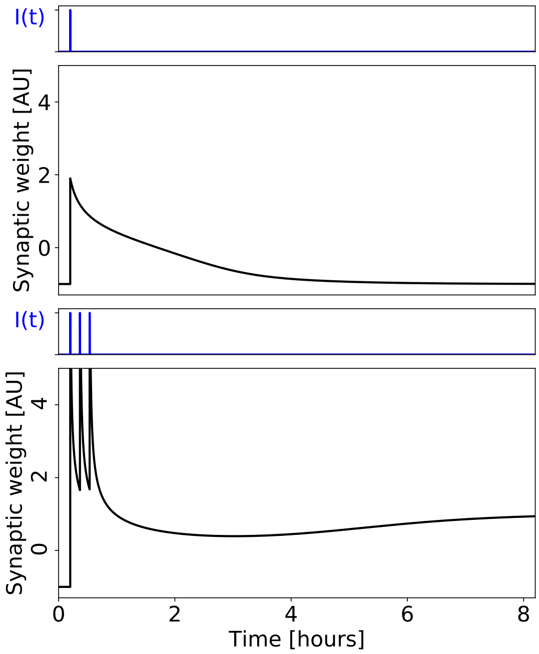

Figure 1: Synaptic weight as a function of time. We try to qualitatively reproduce Fig. 2 of [Frey and Morris, 1997]. In blue the trace is of the input current and in black is the response of the synaptic weight which should be proportional to the change in EPSP in the experimental paper. We considered two stimulation protocols. The first one is one single stimulation pulse, or “weak tetanus , duration 0.2 s. The second in three repeated stimuli, or “strong tetanus: duration 1 s with 10 min intervals.

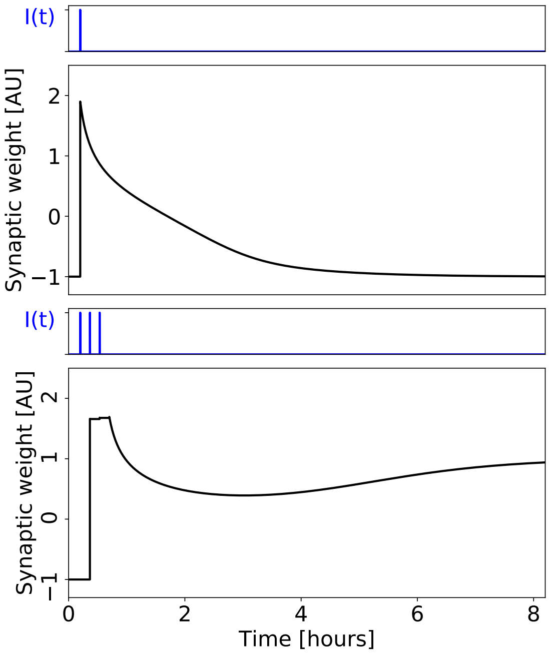

Figure 2: Same as Fig. 1, but after applying a low-pass filter on the value of synaptic weights, so to mimic a saturation effect of the synaptic efficacy.

References

[Frey and Morris, 1997] Frey, U. and Morris, R. G. M. (1997). Synaptic tagging and long-term potentiation. *Nature*, 385:533–536.
